# Genome Sequencing and Assembly Strategies and a Comparative Analysis of the Genomic Characteristics in Penaeid Shrimp Species

**DOI:** 10.3389/fgene.2021.658619

**Published:** 2021-05-03

**Authors:** Jianbo Yuan, Xiaojun Zhang, Fuhua Li, Jianhai Xiang

**Affiliations:** ^1^CAS Key Laboratory of Experimental Marine Biology, Institute of Oceanology, Chinese Academy of Sciences, Qingdao, China; ^2^Laboratory for Marine Biology and Biotechnology, Qingdao National Laboratory for Marine Science and Technology, Qingdao, China; ^3^Center for Ocean Mega-Science, Chinese Academy of Sciences, Qingdao, China

**Keywords:** penaeid shrimp, genome, genome assembly, genomic characteristic, whole genome duplication

## Abstract

Penaeid shrimp (family Penaeidae) represents one of the most economically and ecologically important groups of crustaceans. However, their genome sequencing and assembly have encountered extreme difficulties during the last 20 years. In this study, based on our previous genomic data, we investigated the genomic characteristics of four penaeid shrimp species and identified potential factors that result in their poor genome assembly, including heterozygosity, polyploidization, and repeats. Genome sequencing and comparison of somatic cells (diploid) of the four shrimp species and a single sperm cell (haploid) of *Litopenaeus vannamei* identified a common bimodal distribution of K-mer depths, suggesting either high heterozygosity or abundant homo-duplicated sequences present in their genomes. However, penaeids have not undergone whole-genome duplication as indicated by a series of approaches. Besides, the remarkable expansion of simple sequence repeats was another outstanding character of penaeid genomes, which also made the genome assembly highly fragmented. Due to this situation, we tried to assemble the genome of penaeid shrimp using various genome sequencing and assembly strategies and compared the quality. Therefore, this study provides new insights about the genomic characteristics of penaeid shrimps while improving their genome assemblies.

## Introduction

Penaeid shrimp belongs to Penaeidae, a family of Decapoda (Crustacea), which comprise many aquatic animals with high ecological and economic values, such as the Pacific white shrimp *Litopenaeus vannamei*, Chinese shrimp *Fenneropenaeus chinensis*, giant tiger prawn *Penaeus monodon*, and kuruma prawn *Marsupenaeus japonicus* (Farfante and Kensley, 1997; Wilson et al., 2000; Koyama et al., 2010). These species are the subject of the most important group in fisheries and aquaculture and have therefore attracted considerable research attention (Dall et al., 1990). According to the statistics from the Food and Agriculture Organization of the United Nations (FAO), shrimp and prawn (majorly penaeid shrimp) are the main groups of exported species that account for ∼16% of the total value of internationally traded fishery production in 2018, just less than that of salmon and trout (∼18%) (FAO, 2020). The production of farmed shrimp reached >6 million tonnes in 2018, valued at over US$38 billion. As the most important farmed crustacean species, *L. vannamei* alone contributed 53% of the total farmed crustacean production. Due to their high commercial values, genome-based selective breeding programs have been conducted to ensure sustainable and profitable production.

In addition to economical values, penaeid shrimp also exhibits some special biological features, including complex body plans, and novelties (Farfante and Kensley, 1997), high frequency of intermittent molting (about 50 molts during a lifetime) (Godin et al., 1996), and the fastest nerve signal conducting speed (∼200 ms^–1^) in animals (Fan et al., 1961). However, the detailed mechanisms of these biological features are far from understood. Thus, numerous recent studies have tried to investigate these mechanisms through whole-genome sequencing (WGS) of penaeid shrimp (Yuan et al., 2018, 2021; Zhang et al., 2019; Uengwetwanit et al., 2021).

Due to their importance, decoding the genomes of these penaeid species has attracted global attention. As early as 1997, an international workshop on genome mapping of aquaculture animals was founded, aiming to construct complete genome maps of five economical important organisms, including penaeid shrimp, salmon, catfish, tilapia, and oyster (Alcivar-Warren et al., 1997). The genomes of the other four species have been published earlier before 2016 (Zhang et al., 2012; Berthelot et al., 2014; Lien et al., 2016; Liu et al., 2016). However, due to the high degree of genome complexity, penaeid shrimp has encountered extreme difficulties in genome sequencing and assembly, and the first penaeid shrimp genome was not completed until 2019 (Zhang et al., 2019). Nowadays, only three high-quality genomes of penaeid shrimp have been reported, namely, *L. vannamei*, *F. chinensis*, and *P. monodon* (Zhang et al., 2019; Uengwetwanit et al., 2021; Yuan et al., 2021). The draft assembly of *M. japonicus* is highly fragmented (Yuan et al., 2018). Even for the three high-quality genomes, the contig N50 lengths (<59 Kb) are significantly shorter than many newly published genomes (mostly > 1 Mb) (Shingate et al., 2020; Meyer et al., 2021) and genomes of many other crustaceans, e.g., *Eulimnadia texana* (18.07 Mb) (Baldwin-Brown et al., 2018) and *Portunus trituberculatus* (4.12 Mb) (Tang et al., 2020). Whereas the factors that cause poor assembly of the penaeid shrimp genome are still unclear, although their genomes are available.

In this study, we collected the genome sequencing data of four representative penaeid shrimp species, including *L. vannamei*, *F. chinensis*, *P. monodon*, and *M. japonicus*, and performed genome survey analyses to investigate their genomic characteristics. And then, we used various methods to conduct genome assembly using these sequencing data and tested how much data would be sufficient for the genome assembly. Based on this study, some clues may be provided for the future higher-quality genome assembly of penaeid shrimps.

## Materials and Methods

### Genome Sequencing Data of Penaeid Shrimp

The Illumina paired-end sequencing data of four penaeid shrimp species (*L. vannamei*, *F. chinensis*, *P. monodon*, and *M. japonicus*) were collected from previous studies with the sequencing read length of 150 bp (PRJNA438564, PRJNA627295, PRJNA387410) (Yuan et al., 2018, 2021; Zhang et al., 2019). A total of 361.5 Gb data for *L. vannamei*, 160.9 Gb data for *F. chinensis*, 127.3 Gb data for *P. monodon*, and 127.5 Gb data for *M. japonicus* were collected. The raw sequencing data were trimmed to filter out low-quality data and adapter contaminants by using the NGS QC Toolkit with the parameters of “2 A -c 10” (Patel and Jain, 2012). The PacBio long-read sequencing data of *L. vannamei* and *F. chinensis* were collected from previous studies with the PacBio sequencing read N50 length of 11,205 and 9,813 bp, respectively (PRJNA438564 and PRJNA627295) (Zhang et al., 2019; Yuan et al., 2021). A total of 132.8 Gb PacBio data for *L. vannamei* and 160.3 Gb PacBio data for *F. chinensis* were collected. The final genome assembly sequences of *L. vannamei*, *F. chinensis*, and *P. monodon* were downloaded from the NCBI with the accession number of QCYY00000000, JABKCB000000000, and JABERT000000000, respectively (Zhang et al., 2019; Uengwetwanit et al., 2021; Yuan et al., 2021). These three genome assemblies were all assembled based on PacBio sequencing data. The contig N50 length are 57.65, 58.99, and 45.08 Kb and the scaffold N50 length are 31.300, 28.92, and 44.86 Mb for *L. vannamei*, *F. chinensis*, and *P. monodon*, respectively.

### Genome Survey Analysis

In order to investigate genomic characteristics of penaeid shrimp, a K-mer (K represents the chosen length of substrings)-based genome survey was conducted to estimate the genome size and complexity. Based on the Illumina paired-end sequencing data, the K-mer frequency along the read was calculated (Li et al., 2010b). Jellyfish was used to calculate K-mer depth distribution (Marcais and Kingsford, 2011), which depends on the characteristic of the genome and follows a Poisson’s distribution. Here, *K* = 19 was selected for the survey analysis.

An empirical formula, G = N × (L-K + 1)/(L × M), was used to calculate the genome size (G), where N is the number of K-mers, L is the read length, K stands for the length of K-mer, and M stands for the observed peak of K-mer depth (Li et al., 2010a). The *M* values of the four shrimp species were calculated, namely, *L. vannamei*, *M* = 37; *F. chinensis*, *M* = 66; *P. monodon*, *M* = 43; and *M. japonicus*, *M* = 47. Besides, genome size can be determined using flow cytometry (approximately 1 pg = 978 Mb) (Dolezel et al., 2003). The genome size estimation results of penaeid shrimp species and other decapods were also downloaded from the Animal Genome Size Database^1^. The flow cytometry estimation of the four penaeid shrimp species were included, namely, *L. vannamei*, 2.50 pg; *F. chinensis*, 1.92 pg; *P. monodon*, 2.53 pg; and *M. japonicus*, 2.83 pg. Combining the results above, the genome size of each penaeid shrimp species could be determined. Besides, the heterozygosity and repeat content of penaeid shrimp were estimated based on the K-mer depth distribution using GenomeScope 2.0^2^.

### Evaluation of Genome Duplication Events in *Litopenaeus vannamei*

To test whether penaeid shrimp has underwent whole-genome duplication, a series of analyses were carried out on the genome sequencing data of *L. vannamei*. Firstly, we sequenced the genome of a single sperm cell of *L. vannamei* and compared its K-mer depth distribution with WGS of somatic cells. Sperms were collected from spermatophore of a male *L. vannamei*. After continuous dilution, a single sperm cell was obtained by using a very thin straw under the microscope, and then the genomic DNA of the cell was subjected to PCR-based whole-genome amplification by the MALBAC^®^ Single Cell WGA Kit (Yikon Genomics, Beijing, China). The amplified DNA fragments were directly used for sequencing on Illumina HiSeq2000 platform (Illumina, San Diego, CA, United States). A total of 1.60 Gb single sperm cell sequencing data were generated, and these data were deposited in NCBI SRA database with the accession number SRR13661692. Unlike somatic cells, single sperm cells are haploid and have low heterozygosity. Thus, the K-mer depth distribution of the single sperm cell sequencing will display some differences with that of WGS of somatic cells in the content of heterozygous K-mers. Jellyfish v2.2 was used to clarify all K-mers in single sperm cell genome sequencing, and the depth value of each K-mer was extracted from the K-mer depth distribution of WGS. The percentage of the K-mers in each depth was calculated to draw K-mer depth distribution plot of single sperm cell genome sequencing.

Next, according to previous studies (Berthelot et al., 2014; Xu et al., 2014), the plot of synonymous site divergence values (Ks) of paralogous genes was widely used to identify genome duplication events of *L. vannamei*. The homologous gene pairs were identified by using an all-to-all BLASTP comparison with *E*-value cutoff of 1E-07. The reciprocal best hit homologous gene pairs were selected to calculate Ks values using the CodeML program from the PAML package (Yang, 2007). The homologous pairs were aligned by MUSCLE (Edgar, 2004), and the well-aligned regions were extracted with Gblocks v0.91b (Talavera and Castresana, 2007).

In addition, the allele frequency distribution was calculated to identify genome duplication events (Pelin et al., 2015). All the Illumina sequencing reads were mapped to the *L. vannamei* genome using Burrows–Wheeler Aligner (BWA) (Li and Durbin, 2009), and all single-nucleotide polymorphisms (SNPs) were called by SAMTools-1.11 (Li et al., 2009). For each site of the SNP, the percentages of the four bases were calculated and sorted from most to least. Then, the allele frequency distribution was calculated based on these percentage values.

### Repeat Annotation

The repeat annotation was performed on four genomes of penaeid shrimp species, *L. vannamei*, *F. chinensis*, *P. monodon*, and *M. japonicus*. Different from the other three species that assembled based on PacBio sequencing data, the *M. japonicus* genome was assembled based on Illumina sequencing data (Yuan et al., 2018). Both RepeatModeler v2.0^3^ and RepeatMasker v4.1.0 were used for *de novo* identification of repeats. A local repeat database was constructed by RepeatModeler, and then, RepeatMasker was used to identify the transposable elements (TEs) by aligning the genome sequences against the local library and RepBase (RepBase21.04) with default parameters (Tarailo-Graovac and Chen, 2009). SciRoKo v3.4 was used to annotate simple sequence repeats (SSRs) in the three penaeid genomes (Kofler et al., 2007).

### Genome Assembly and Comparison

Based on the Illumina sequencing data, the draft genomes of the four penaeid shrimp species (*L. vannamei*, *F. chinensis*, *P. monodon*, and *M. japonicus*) were assembled by SOAPdenovo2 with the *k* value set from 31 to 99 (Luo et al., 2012). Besides, the SOAPdenovo2 assembly was also performed on different amounts of sequencing data (genome coverage of 16 × to 135 ×) of *L. vannamei*.

Based on the PacBio sequencing data, various assembly approaches were used for the genome assembly of *L. vannamei* and *F. chinensis*, including FALCON v0.3.0 (Chin et al., 2016), HABOT2 (Zou et al., 2017), DBG2OLC (Ye et al., 2016), SMARTdenovo (Liu et al., 2020), and WTDBG2 (Ruan and Li, 2020). Due to the lack of raw PacBio sequencing data, genome assembly of *P. monodon* and *M. japonicus* have not been conducted using these assemblers. For FALCON assembly, the long sequencing subreads were firstly selected as the seed reads to be corrected by short subreads, and then, the error-corrected reads were assembled into contigs using FALCON with the parameters of “seed_coverage = 30, length_cutoff_pr = 1,000, length_cutoff = −1.” For HABOT2 assembly, three main modules, namely, graph module, align module, and *Denovo* module, were used to get a hybrid assembly of the subreads with the parameters of “-k 17 -i 1 -m 3 -s 1.” For DBG2OLC assembly, both long PacBio subreads and contigs obtained from a de Bruijn graph (DBG) assembly were used for genome assembly with the parameters of “k 17 MinOverlap 20 AdaptiveTh 0.01 Remove Chimera 1.” The contigs are generated from SOAPdenovo assembly of Illumina sequencing data. For SMARTdenovo assembly, the raw PacBio sequencing subreads were directly used for the assembly follows the overlap-layout-consensus (OLC) paradigm with the parameters of “-c 1.” For WTDBG2 assembly, the subreads were chopped into 1,024-bp segments, similar segments were merged into a vertex, and vertices were connected based on the segment adjacency on subreads. Since WTDBG2 had a better performance in penaeid shrimp genome assembly than the other four methods, it was used for the assembly of different amounts of PacBio sequencing data (genome coverage of 20× to 70×) of *L. vannamei*.

### Quality Assessment of Genome Assembly

To evaluate the quality of the genome assemblies of the penaeid shrimp species in this study and those published in previous studies (Zhang et al., 2019; Uengwetwanit et al., 2021; Yuan et al., 2021), several approaches were utilized to identify the completeness and accuracy of these assemblies. Firstly, Illumina sequencing reads were mapped back to the genome using Bowtie2 with the following parameters: –rdg 3,1 –rfg 3,1 –gbar 2, and the mapping rates were calculated (Langmead and Salzberg, 2012). Besides, according to previous study (Yuan et al., 2020), the unigenes that assembled from the transcriptome data were also mapped to the shrimp genomes using BLAT (Kent, 2002). The unigenes were downloaded from the shrimp gene database^4^ with N50 lengths ranging from 1.40 to 2.34 Kb. In addition, BUSCO v4.0 tool suite was used to evaluate the quality of the genome assemblies by calculating the coverage of the eukaryotic single-copy core genes (BUSCOs, Eukaryota *odb9*) (Seppey et al., 2019).

### Statistical Methods

The statistics for this study are conducted using Student’s *t* test (between two groups) and one-way ANOVA (among three or more groups) using SPSS 22.0 software^5^. Significant differences are indicated when *p* value < 0.05.

## Results

### Genome Survey of Penaeid Shrimp Species

**FIGURE 1:**
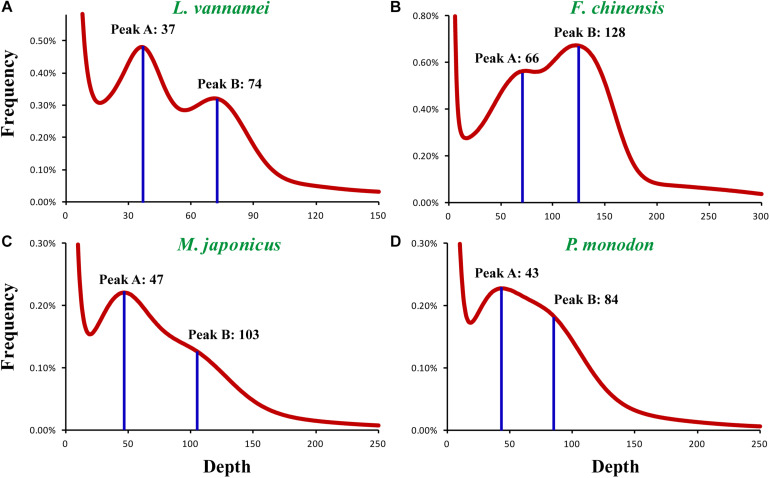
K-mer distribution of four penaeid shrimp genomes. The K-mer distribution plots of the four shrimp species include **(A)** *L. vannamei*, **(B)** *F. chinensis*, **(C)** *M. japonicus*, and **(D)** *P. monodon*. *K* = 19 was selected for the K-mer frequency statistics.

In order to find the factors resulting in the poor assembly of the penaeid shrimp genomes, a comprehensive study of the general genomic characteristics, including genome size, heterozygosity, and repeat content, was conducted on these species. A K-mer-based genome survey was performed on the Illumina sequencing data of four representative penaeid shrimp species, *L. vannamei*, *F. chinensis*, *P. monodon*, and *M. japonicus*. Two peaks (Peak A and Peak B) were detected in the K-mer plot of all the four species (Figure 1), and the K-mer depth of Peak B was about twice of that for Peak A, e.g., K-mer depth of Peak A and Peak B were 37 and 74 in *L. vannamei*, respectively. Generally, according to previous studies (Zhang et al., 2012; Li et al., 2017; Shingate et al., 2020), Peak A represents the heterozygous single copy K-mers, while Peak B represents the homozygous single copy K-mers in the genome, which is also used for genome size estimation. However, the genome size estimated based on the K-mer depth of Peak B was half of that based on Peak A and also half of that estimated by flow cytometry methods (Alcivar-Warren et al., 1997; Zhang et al., 2019). Thus, it was confusing about which peak represents the homozygous single copy K-mers.

### Genome Sizes of Penaeid Shrimp Species

**FIGURE 2:**
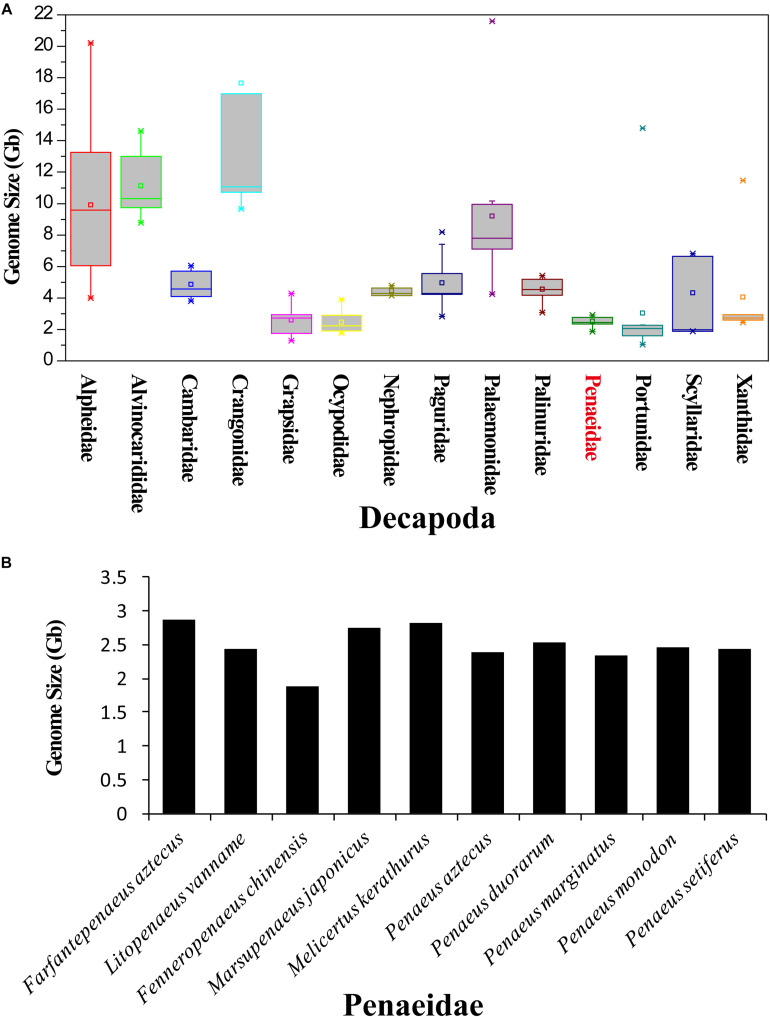
Genome size of decapods and various penaeid shrimp species. **(A)** Genome sizes of various families of Decapoda. The information of genome sizes was obtained from the Animal Genome Size Database (www.genomesize.com). **(B)** Genome sizes of various penaeid shrimp species.

According to the Animal Genome Size Database, the genome sizes of 145 decapods, covering 32 families, were recorded. Among them, the largest genome was 39.87 Gb (*Sclerocrangon ferox*) and the smallest genome was 1.04 Gb (*Carcinus maenas*). Genomes from Alpheidae (9.92 ± 4.99 Gb), Alvinocarididae (11.12 ± 2.04 Gb), Crangonidae (17.66 ± 12.73 Gb), and Palaemonidae (9.21 ± 4.98 Gb) have relatively larger sizes (Figure 2A). The genome sizes of Portunidae (1.86 ± 0.44 Gb, excluding *Necora puber*, as it has a singular genome size of 14.79 Gb), Penaeidae (2.51 ± 0.29 Gb), and Ocypodidae (2.45 ± 0.73 Gb) were smaller than those of other families.

The genome sizes of various penaeid shrimp species (Penaeidae) were around 2.5 Gb (Figure 2B). *Farfantepenaeus aztecus* has been identified to have the largest genome of 2.87 Gb, and the genome of *F. chinensis* was identified to be the smallest (1.87 Gb) among these penaeids. After combining the results of flow cytometry and K-mer analysis (Peak A), the genome sizes of the four penaeid shrimp species were estimated, namely, *L. vannamei*, 2.45 Gb; *F. chinensis*, 1.88 Gb; *P. monodon*, 2.66 Gb; and *M. japonicus*, 2.38 Gb.

### Genome Duplication Evaluation

Generally, penaeid shrimp were considered diploid, which is supported by their karyotypes (Campos-Ramos, 1997; Mansouri et al., 2011). Here, we adopted a series of approaches to identify whether penaeid shrimp has undergone whole-genome duplication based on the genome data of *L. vannamei*.

**FIGURE 3:**
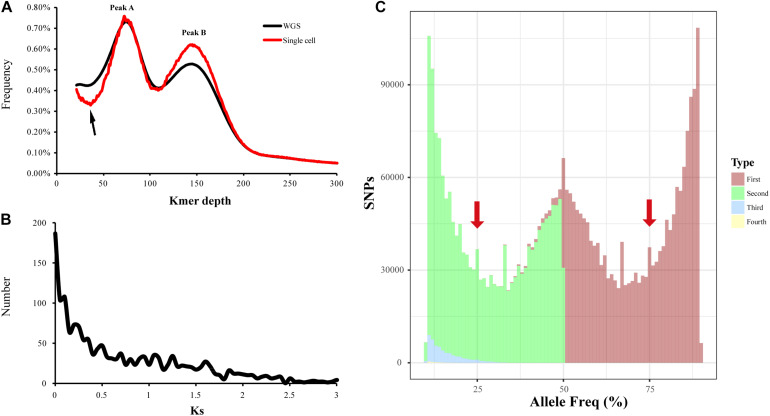
Evaluation of genome duplication of *L. vannamei*. **(A)** K-mer distribution of the single sperm cell and whole-genome sequencing (WGS) data. **(B)** Ks frequency distribution for pairs of paralogous genes in the *Litopenaeus vannamei* genome. **(C)** Allele frequency spectra based on read counts of single-nucleotide polymorphisms (SNPs). For each SNP site, the appearance frequencies of the four bases were sorted from most (Type: First) to least (Type: Fourth). Leftmost and rightmost truncated peaks likely correspond to variations between individuals in a population. For polyploidy genome, the peaks at 25, 50, and 75% would be expected.

Firstly, in order to identify which peak (Peak A or Peak B) represents homozygous K-mers, we sequenced the genome of a single sperm cell (haploid) of *L. vannamei* and compared the K-mer plot with that of WGS of somatic cells (diploid). Unexpectedly, the two peaks were also found in the K-mer plot of single cell sequencing, and Peak A highly fitted with that of WGS (Figure 3A). Besides, a lower trough was detected in front of Peak A, indicating the lower heterozygosity of the single sperm cell than somatic cells. Therefore, this result supported that Peak A represents the homozygous K-mers, and lots of genomic segments might be duplicated. As for the Peak B, the peak detected in the K-mer plot of single cell sequencing was higher than that of WGS, which may be due to the higher chance to be amplified and sequenced for duplicated sequences than homozygous sequences.

Ks analysis of the homologous gene pairs was also used to identify whole-genome duplication. Approximately 3,276 reciprocal best hit paralogous genes were identified in the *L. vannamei* genome, and the Ks values of these gene pairs were calculated. The spectrum of Ks showed L-shaped distribution with no obvious peak, indicating that the penaeid shrimp may not have undergone whole-genome duplication (Figure 3B).

Additionally, the allele frequency distribution of penaeid shrimp was calculated and compared with those of polyploid species (Pelin et al., 2015). After mapping the Illumina sequencing reads to the *L. vannamei* genome, a total of 4,185,110 SNPs were called. It was found that the allele frequency plot of all SNPs followed a unimodal distribution, with a peak at 0.5 (Figure 3C). The leftmost and rightmost truncated peaks may correspond to the variation between individuals in a population. According to previous study (Pelin et al., 2015), no peaks should be observed in haploids, unimodal distributions should be expected for diploids, and non-random trimodal distribution can be observed in polyploids. Unlike those of polyploidy genomes, peaks at 0.25 and 0.75 were not detected in the allele frequency plot of *L. vannamei*, but a unimodal distribution was found, suggesting that penaeid shrimp is diploid.

Overall, we speculated that penaeid shrimp was diploid without whole-genome duplication. Peak A in the K-mer plots represented the homozygous K-mers, thus, there might be a large amount of repetitive sequences in the penaeid shrimp genomes, and relatively low heterozygosity was expected. The heterozygosity of *L. vannamei*, *P. monodon*, and *M. japonicus* was estimated to be 0.26, 0.21, and 0.19% (model fit values ranged from 88.33 to 95.99%), respectively.

### Repeats in the Penaeid Shrimp Genomes

The repeats were annotated in the genomes of four penaeid shrimp species that were published in previous studies, *L. vannamei*, *F. chinensis*, *P. monodon*, and *M. japonicus*. According to these studies (Yuan et al., 2018, 2021; Uengwetwanit et al., 2021), the first three genomes were assembled based on PacBio sequencing data, while the *M. japonicus* genome was assembled based on Illumina sequencing data, as no PacBio data were available. Repeats accounted for about 50% of the first three genomes, and the amount of TEs were varied among them that *L. vannamei* contained the least TEs (16.25%) and *P. monodon* contained the most TEs (22.01%) (Table 1). DNA transposons were highly expanded in the genomes of *L. vannamei* (9.33%) and *F. chinensis* (9.33%) compared to those in *P. monodon* (5.87%) and *M. japonicus* (5.66%) (*p* < 0.05). Whereas in the *P. monodon* genome, long interspersed nuclear elements (LINEs) were the most abundant TEs (9.26%) that was significantly higher than those in *L. vannamei* (2.82%), *F. chinensis* (3.27%), and *M. japonicus* (4.75%) (*p* < 0.05). Besides, short interspersed nuclear elements (SINEs) and long terminal repeats (LTRs) were also abundant in *P. monodon*.

**TABLE 1:** Summary of repetitive sequences in four penaeid shrimp genomes.

**Repeats**	***L. vannamei***	***F. chinensis***	***P. monodon***	M. japonicus*
Genome length	1.66 Gb	1.57 Gb	2.39 Gb	1.79 Gb
Total repeats	49.39%	48.58%	42.83%	34.96%
DNA	9.33%	13.00%	5.87%	5.66%
LINE	2.82%	3.27%	9.26%	4.75%
SINE	0.06%	0.11%	1.30%	0.03%
LTR	0.62%	0.53%	1.42%	1.14%
Unknown	3.42%	3.52%	4.16%	7.19%
Satellite	0.10%	0.16%	0.00%	0.35%
Simple repeats	23.93%	19.50%	15.01%	9.79%
Low complexity	9.49%	8.49%	5.81%	6.28%

*The repeat annotation of *Marsupenaeus japonicus* was based on the assembly of Illumina sequencing data, and the annotation of the other three species were based on the genome assemblies published in previous studies (Zhang et al., 2019; Uengwetwanit et al., 2021; Yuan et al., 2021). LINE, long interspersed nuclear element; LTR, long terminal repeat; SINE, short interspersed nuclear element.

**FIGURE 4:**
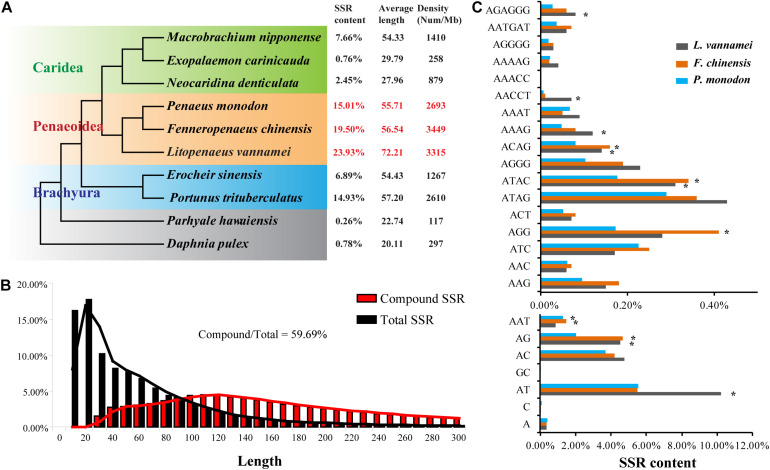
Simple sequence repeats (SSRs) in penaeid shrimp genomes. **(A)** Content, average length, and density of SSRs in the genomes of various crustaceans. The phylogenetic tree was referred from a previous study (Yuan et al., 2018). The characteristics of SSRs in various crustaceans were referred from the study of Yuan et al. (2021). Since the SSR content may be underestimated in the *Marsupenaeus japonicus* genome, it was not shown and compared with the other three shrimp genomes. **(B)** The length distribution of compound SSRs and total SSR in the *Litopenaeus vannamei* genome. The compound SSR indicates numerous different types of SSRs connected head to tail. **(C)** Comparisons of the distributions of various types of SSRs in three penaeid shrimp genomes. A part of SSR types with relatively high content in the genome was shown in the plot.

Besides TEs, SSRs were also abundant in the first three shrimp genomes, *L. vannamei*, 23.93%; *F. chinensis*, 19.50%; and *P. monodon*, 15.01%), which have been identified as the most abundant among the species whose genomes are available (Zhang et al., 2019). As assembled based on Illumina sequencing data, the SSR content was possibly underestimated in the *M. japonicus* genome (9.79%). Similar results have been identified in the Illumina sequencing data assembly of *L. vannamei* (10.33%), *F. chinensis* (9.03%), and *P. monodon* (10.90%). When comparing with other decapods, the content of SSRs of penaeid shrimp were more abundant (*p* < 0.05), with significantly higher density (2,693–3,449 per Mb) and similar length distribution (56.54–72.21 bp in average) (Figure 4A). SSRs were densely distributed in the penaeid shrimp genomes, and thus, a large amount of compound SSRs, which are composed of different types of SSRs that linked head to tail, have been identified in these genomes. Among the total SSRs, approximately 60% of them were identified to form compound SSRs, which were significantly higher than those in many other crustaceans (<24%; Supplementary Figure 1). Besides, the lengths of compound SSRs were significantly longer than those of single SSRs (*p* < 0.05) (Figure 4B).

Except for (GC)_n_, dinucleotide SSRs [(AT)_n_, (AC)_n_, (AG)_n_] were the most abundant SSRs in the penaeid shrimp genomes, which accounted for more than 73% of total SSRs (Figure 4C). The SSR compositions were quite similar among the three shrimp species, whereas some variations were also observed. *L. vannamei* had significantly higher amounts of (AT)_n_ and (AACCT)_n_ than those of the other two species, while *F. chinensis* and *P. monodon* had significantly higher amounts of (AG)_n_, (AAT)_n_, (ATAC)_n_, and (ACAG)_n_ than those of *L. vannamei*.

### Genome Assembly of Penaeid Shrimp Species

**FIGURE 5:**
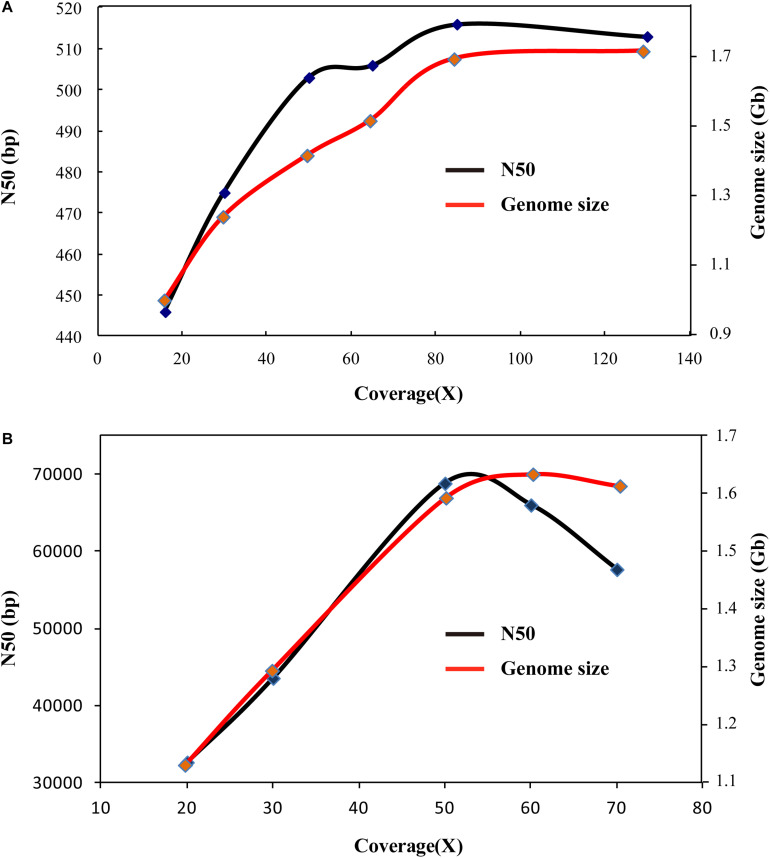
Genome assembly using different coverages of sequencing data. **(A)** Genome assembly comparison using different coverages of Illumina sequencing data. **(B)** Genome assembly comparison using different coverages of PacBio sequencing data.

Based on various sequencing data of penaeid shrimp, various genome assembly strategies have been carried out on these shrimp species. Firstly, based on the Illumina sequencing data, SOAPdenovo assembly was performed on the four penaeid shrimp species, *L. vannamei*, *F. chinensis*, *P. monodon*, and *M. japonicus*. However, these assemblies were rather poor in quality, similar to many previous studies (Yu et al., 2015; Yuan et al., 2018). The contig N50 lengths ranged from 301 bp (*P. monodon*) to 514 bp (*L. vannamei*) (Supplementary Table 1), which indicated that these assemblies were highly fragmented. Besides, after extending the contigs by filling gaps in scaffolds that assembled based on large insert sequencing libraries (insert size of 2, 5, and 10 Kb and read length of 100 bp, PRJNA438564), the contig N50 length could only reach 2.8 Kb in *L. vannamei* (Table 2). In addition, we performed genome assembly based on various amounts of sequencing data (16 ×–135 ×). When the sequencing depth reached 80 ×, the genome assembly size and N50 length tend to be stable (Figure 5A), and the assembly showed high completeness that covered more than 91% of the transcriptome unigenes. It indicated that the Illumina sequencing data are sufficient for assembly, while the poor assembly might be caused by the high complexity of the genome and/or the limitation of a short sequencing read length (150 bp).

**TABLE 2:** Statistics of genome assembly of *Litopenaeus vannamei* using different methods.

	**SOAPdenovo**^§^	**FALCON**	**HABOT2**	**DBG2OLC**	**SMARTdenovo**	**WTDBG2**
Contig number	982,421	463,151	110,906	43,938	60,355	50,304
Total length (Gb)	1.35	1.59	1.69	1.30	1.78	1.62
Longest (Kb)	1,219	1,219	214	707	422	739
N50 (bp)	2,826	9,496	25,477	43,564	34,826	57,650
N90 (bp)	712	1,271	9,552	13,276	15,383	14,641
Unigene coverage	95.76%	89.56%	93.73%	83.16%	68.53%	94.45%
Unigene coverage (50%)*	85.50%	78.33%	84.85%	71.24%	50.12%	86.91%

^§^ SOAPdenovo genome assembly was conducted based on the Illumina sequencing data. * Unigene coverage (50%) indicates more than 50% of a unigene sequence covered by a single scaffold.

As for the assembly of PacBio sequencing data, various amounts of the data (20 ×–70 ×) of *L. vannamei* were used to test the adequacy of the data first. When the sequencing data coverage reached 50 ×, the total length of the assembly tended to be stable, but when the sequencing coverage reached 70 ×, the N50 length became shorter (Figure 5B). However, the assembly of 70 × data showed higher completeness (94.45%) than that of 60 × (89.65%) and 50 × data (82.59%). Thus, 70 × PacBio sequencing data are sufficient for the genome assembly. As we only collected PacBio sequencing data of *L. vannamei* and *F. chinensis* in our previous studies (Zhang et al., 2019; Yuan et al., 2021), we only performed genome assembly of these two species herein. Based on the total PacBio sequencing data, we assembled the *L. vannamei* and *F. chinensis* genomes using five methods, namely, FALCON, HABOT2, DBG2OLC, SMARTdenovo, and WTDBG2. Except for DBG2OLC, the total length of the assemblies by the other four methods was about 1.6 Gb in *L. vannamei* (Table 2). Different from the assembly based on Illumina sequencing data, the contigs assembled based on PacBio sequencing data showed significantly higher continuity (*p* < 0.05). In the two shrimp species, the contig N50 length was at least three times longer than the SOAPdenovo assembly (Table 2; Supplementary Table 2). The N50 length of the WTDBG2 assembly even reached 57,650 bp in *L. vannamei* and 58,996 bp in *F. chinensis*, which was more than 20 times longer than that in the SOAPdenovo assembly. Besides, WTDBG2 assembly not only has higher continuity than other methods but also has higher completeness (covering more than 94% of unigenes).

Although the assembly of PacBio sequencing data has higher continuity than that of SOAPdenovo assembly, it was still highly fragmented, as it was composed of more than 40,000 contigs, and the contig N50 lengths were significantly shorter than many recently published crustacean genomes, e.g., *Eulimnadia texana* (18.07 Mb) (Baldwin-Brown et al., 2018), *P. trituberculatus* (4.12 Mb) (Tang et al., 2020), and *Paralithodes platypus* (147.47 Kb) (Tang et al., 2021). Thus, we next investigated the factors that caused the high fragmentation of these assemblies. As for SOAPdenovo assembly, we mapped the contigs and Illumina sequencing reads on a complete bacterial artificial chromosome (BAC) (SHE003C23), which was previously sequenced by the Sanger sequencing platform (Zhang et al., 2010, 2019). Low coverage of Illumina sequencing data was found in many regions, which was consistent with the lack of contigs in these regions (Supplementary Figure 2). When analyzing these low coverage regions, it was found that they were mainly composed of SSRs. Especially for the regions with extremely long single or compound SSRs, almost no sequencing reads were distributed in these regions. The read coverage of SSR regions (20.42 ×) was significantly lower than those of TE (190.32 ×) and other regions (202.98 ×) (*p* < 0.05) (Supplementary Figure 3).

As for the PacBio data assembly, we also mapped the assembly contigs to the sequenced BACs to find the factors that result in the assembly fragmentation. However, these BACs were aligned to the contigs in full length (Zhang et al., 2019), and thus the characteristics of the edges of these contigs could not be identified.

## Discussion

The study of the penaeid shrimp genome is attractive globally due to its high economic and biological values. Although several penaeid shrimp genomes have been published (Zhang et al., 2019; Uengwetwanit et al., 2021; Yuan et al., 2021), the factors that cause genome assembly difficulties and poor assembly quality are still ambiguous. In this study, two aspects have been identified to be the potential causes for these problems. The first one was the high percentage of homo-duplicated repeats or high heterozygosity. Two peaks were identified in the K-mer depth distribution plots of all the four penaeid shrimp species, which were similar to those genomes with high heterozygosity, e.g., the Pacific oyster *Crassostrea gigas* (Zhang et al., 2012) and the Zhikong scallop *Chlamys farreri* (Li et al., 2017). Even for the genomes that underwent whole-genome duplication, e.g., the horseshoe crab *Limulus polyphemus* (Nossa et al., 2014) and the pineapple *Ananas comosus* (Ming et al., 2015), the former peak of the two peaks in the K-mer plots also represented heterozygous K-mers. If Peak A represents heterozygous K-mers, penaeid shrimp will have a high degree of heterozygosity that was estimated to be 2.43% in *L. vannamei*, 1.95% in *F. chinensis*, 4.95% in *P. monodon*, and 4.49% in *M. japonicus*. However, the results of genome size estimation and K-mer depth distribution of single sperm cell sequencing supported Peak A that represents homozygous K-mers, while Peak B represents homo-duplicated K-mers. Whereas no signature of whole-genome duplication has been identified in the penaeid shrimp genomes through Ks and allele frequency analyses. Furthermore, a single Hox gene cluster was identified in the penaeid shrimp genomes (Yuan et al., 2018; Zhang et al., 2019; Uengwetwanit et al., 2021), which also did not support the whole-genome duplication event. No matter what Peak A represents, the high heterozygosity and homo-duplication both will be responsible for a large number of polymorphic sites in genome sequencing, which will make the genome assembly very difficult.

The abundant SSRs in the penaeid shrimp genome appear to be the second aspect resulting in the poor assembly. In most sequenced species, SSRs only account for ∼1% of the genome (Oliveira et al., 2006; Zhang et al., 2019), whereas the penaeid shrimp genome is particularly notable for having the highest proportion of SSRs (>15%) among sequenced animal genomes up to now. Low coverage of Illumina sequencing data was detected around the SSR regions, which makes the SOAPdenovo assembly highly fragmented. Thus, no matter how much Illumina data are sequenced, the contig N50 lengths of these penaeid shrimp species were very short due to the assembly blocks at the edges of the SSR regions. Even though the third-generation sequencing could cover most of the SSR regions, the large number of SSRs also brings great difficulties to genome assembly. SSRs could be linked head to tail to form a compound SSR, which is much longer than a single SSR. The extremely long compound SSRs will also result in the blocks of the assembly based on PacBio sequencing data. Besides, the OLC paradigm and the DBGs are two major algorithms that are widely used in many genome assembly methods (Ruan and Li, 2020). Both algorithms need to perform sequence mapping and selecting the best hits for the assembly, whereas the simple composition of SSRs will make these processes more difficult. Therefore, even if the PacBio sequencing data are sufficient or excessive for genome assembly of the penaeid shrimp, the contig N50 length has not increased in expectation. Although it is still unclear what results in the fragmentation of the assemblies based on PacBio sequencing data, the sequences in the gaps between contigs will be more complex than we thought. And there may be many other potential factors affecting the genome assembly of penaeid shrimp, which need further investigation.

Before the development of PacBio sequencing technology, Illumina sequencing was widely used for most genome assemblies. As expected, the performance of the Illumina data assembly was worse than that of PacBio data in penaeid shrimp species. However, Illumina sequencing is still used for genome assembly in recent years (Li et al., 2017; Leclere et al., 2019) and also widely used for whole-genome resequencing nowadays. Since the development of the third-generation sequencing technology, many methods for genome assembly have been developed. Finding an effective method to assemble the target genome assembly is undoubtedly important. FALCON has been widely and firstly selected for the genome assembly of most species, whereas it seems unsuitable for the penaeid shrimp genome assembly because of its poor assembly results and extraordinarily long time for the error correction before the assembly. Similar assembly results were obtained through using HABOT2, DBG2OLC, SMARTdenovo, and WTDBG2, but the assembly quality of WTDBG2 was the highest. Thus, WTDBG2 was ultimately used for the genome assembly of the three penaeid shrimp species *L. vannamei*, *F. chinensis*, and *P. monodon* (Zhang et al., 2019; Uengwetwanit et al., 2021; Yuan et al., 2021). The final assembly of *L. vannamei* and *F. chinensis* was similar (Zhang et al., 2019; Yuan et al., 2021). The length of contig N50 was about 58 Kb, which was also similar to that of *P. monodon* (45 Kb) (Uengwetwanit et al., 2021). These three genomes showed high completeness that the coverages of unigenes, Illumina sequencing reads, and BUSCOs were all higher than 91% (Supplementary Table 3). Besides, in order to assemble genome into chromosomal level, Hi-C data were used for scaffolding the contigs of these shrimp species (2n = 88 chromosomes). Finally, these contigs were anchored onto 44 chromosomes, and their scaffold N50 lengths ranged from 30 to 45 Mb.

Besides the PacBio continuous long-read (CLR) sequencing, there are many other long-read sequencing technologies, such as Oxford Nanopore Technologies (ONT) (Feng et al., 2015). The ONT sequencing can generate long reads, with an average length of more than 40 Kb, which is 2–4 times longer than that of PacBio sequencing (Lang et al., 2020). The assembly based on longer sequencing reads will assemble longer contigs; thus, we have tried to conduct ONT sequencing on *L. vannamei*. However, due to the limitation of data generation and short sequencing reads, the ONT sequencing of penaeid shrimp failed. There are many other assembly methods that were not used herein, and they may also be suitable for genome assembly of penaeid shrimp species. For chromosomal-level assembly, besides Hi-C sequencing, Bionano genome mapping also supports individual chromosome physical mapping and assembly in complex genomes (Stankova et al., 2016). Further research on the strategies of genome sequencing and assembly will aid the construction of high-quality genomes of penaeid shrimp. Furthermore, with the development of new sequencing technologies and assembly methods, higher-quality genome assemblies of peaneid shrimp species can be obtained in the future. This study can provide some clues for the future genome assembly of penaeid shrimp species.

## Data Availability Statement

The original contributions presented in the study are included in the article/Supplementary Material, further inquiries can be directed to the corresponding author.

## Author Contributions

JX and FL conceived and designed the study. JY conducted the genome assembly and bioinformatics analyses. XZ performed genome sequencing. JY wrote the manuscript. XZ revised the manuscript. All authors read and approved the final manuscript.

## Conflict of Interest

The authors declare that the research was conducted in the absence of any commercial or financial relationships that could be construed as a potential conflict of interest.

## Footnotes

1 www.genomesize.com

2 http://qb.cshl.edu/genomescope/

3 http://www.repeatmasker.org/RepeatModeler.html

4 http://www.genedatabase.cn/Decapoda.html

5 https://www.ibm.com/analytics/spss-statistics-software

## Supplementary Material

Supplementary Figure 1 Compound SSR length distribution in four crustaceans.

Supplementary Figure 2 The coverage of Illumina sequencing reads and contigs in a complete BAC (SHE003C23).

Supplementary Figure 3 The comparison of read coverages of various genomic regions.

Supplementary Table 1 SOAPdenovo assembly of four penaeid shrimp species.

Supplementary Table 2 Statistics of genome assembly of *F. chinensis* using different methods.

Supplementary Table 3 Statistics of genome assembly of four penaeid shrimp species.
