# Supplementary material for: Genome Sequencing and Assembly Strategies and a Comparative Analysis of the Genomic Characteristics in Penaeid Shrimp Species
Source: Front Genet. 2021 May 3;12:658619. doi: 10.3389/fgene.2021.658619 (PMC8126689; doi:10.3389/fgene.2021.658619)
Supplement: Supplementary Figure 1 — Compound SSR length distribution in four crustaceans. [file Data_Sheet_1.docx]

Supplementary Material

For

Genome Sequencing and Assembly Strategies and a Comparative Analysis of the Genomic Characteristics in Penaeid Shrimp Species

**Jianbo Yuan, Xiaojun Zhang, Fuhua Li, Jianhai Xiang**

# Supplementary Tables

**Table S1.** SOAPdenovo assembly of four penaeid shrimp species.

|  | ***L. vannamei*** | ***F. chinensis*** | ***P. monodon*** | ***M. japonicus*** |
| --- | --- | --- | --- | --- |
| Number: | 3,551,678 | 2,745,701 | 7,106,289 | 5,632,117 |
| Total length (bp): | 1742739422 | 1,119,167,375 | 1,882,378,599 | 1,924,054,682 |
| Longest (bp): | 94,237 | 18,658 | 12,599 | 16,221 |
| Shortest (bp): | 100 | 100 | 100 | 100 |
| N50 (bp): | 514 | 504 | 301 | 416 |
| N90 (bp): | 187 | 196 | 138 | 159 |

**Table S2. Statistics of genome assembly of *F. chinensis* using different methods.**

|  | **SOAPdenovo^§^** | **FALCON** | **HABOT** | **DBG2OLC** | **Smartdenovo** | **WTDBG** |
| --- | --- | --- | --- | --- | --- | --- |
| Contig number: | 2,674,510 | 673,265 | 23,223 | 43,686 | 172,081 | 41,848 |
| Total length: | 1,198,038,017 | 1,344,294,307 | 1,371,654,560 | 1,399,189,217 | 1,813,402,606 | 1,554,960,535 |
| Longest: | 37,524 | 273,274 | 945,713 | 522,485 | 82,430 | 829,035 |
| N50: | 809 | 10,396 | 21,818 | 46,586 | 11,698 | 58,996 |
| N90: | 176 | 536 | 2,584 | 14,474 | 6,695 | 13,999 |
| Unigene coverage | 80.50% | 89.48% | 84.61% | 84.32% | 79.03% | 94.83% |
| Unigene coverage (50%)* | 63.14% | 78.93% | 68.49% | 71.78% | 41.06% | 84.31% |

**^§^** SOAPdenovo genome assembly was conducted based on the Illumina sequencing data. * Unigene coverage (50%) indicates more than 50% of a unigene sequence covered by a single scaffold.

**Table S3. Statistics of genome assembly of four penaeid shrimp species. ^§^**

| **Species** | ***L. vannamei*** | ***F. chinensis*** | ***P. monodon*** | ***M. japonicus*^＄^** |
| --- | --- | --- | --- | --- |
| **Contigs** | | | |  |
| Total length (bp) | 1,618,026,442 | 1,554,960,535 | 2,000,783,471 | 1,924,054,682 |
| Longest (bp) | 739,419 | 829,035 | 1,387,722 | 16,221 |
| N50 (bp) | 57,650 | 58,996 | 45,084 | 416 |
| N90 (bp) | 14,641 | 13,999 | 916 | 159 |
| **Scaffolds** | | | |  |
| Total length (bp) | 1,631,536,563 | 1,581,129,620 | 2,394,347,767 | 1,942,550,811 |
| Longest (bp) | 47,298,368 | 45,805,217 | 65,869,259 | 1,606,464 |
| N50 (bp) | 31,296,514 | 28,916,617 | 44,862,054 | 937 |
| N90 (bp) | 63,975 | 66,757 | 25,075 | 189 |
| **Quality evaluation** | | | |  |
| Unigene coverage | 94.45% | 94.83% | 96.81% | 98.63% |
| Unigene coverage (50%)* | 86.91% | 84.31% | 93.79% | 82.39% |
| Illumina reads coverage | 93.25% | 91.12% | 93.00% | 94.05% |
| BUSCO | 93.13% | 92.69% | 94.72% | 82.66% |

**^§^** This table summarized the four genome assemblies in previous studies ([Zhang et al., 2019](#_ENREF_56);[Uengwetwanit et al., 2021](#_ENREF_44);Yuan et al., 2021;Yuan et al., 2018). * Unigene coverage (50%) indicates more than 50% of a unigene sequence covered by a single scaffold**^＄^**. Genome assembly of *M. japonicus* was based on the Illunima sequencing data.

# Supplementary Figures


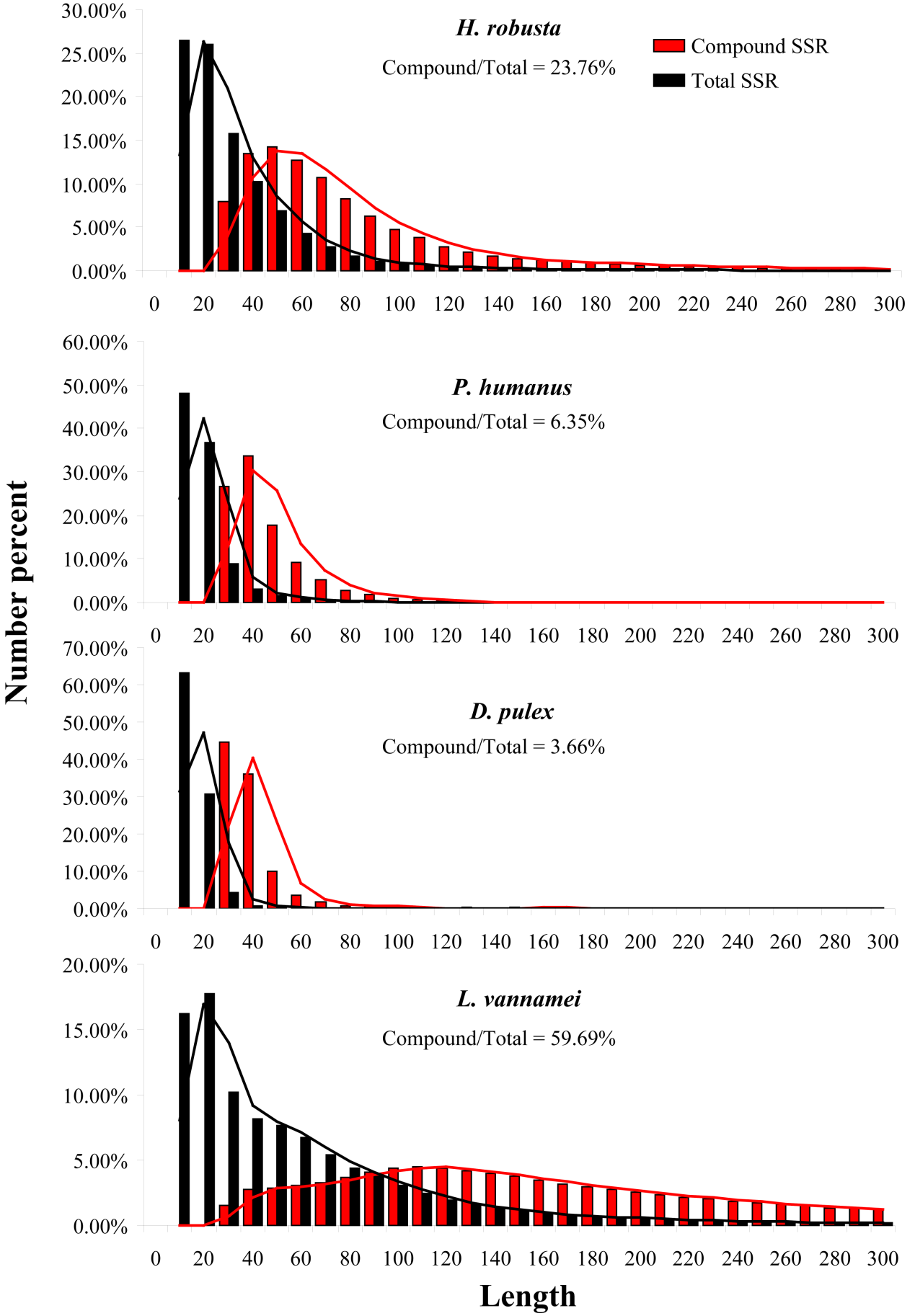


**Fig. S1.** Compound SSRs length distribution in four crustaceans.


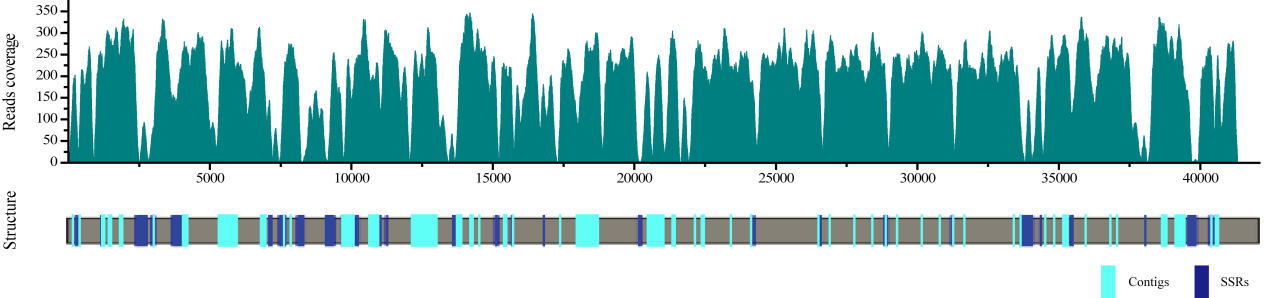


**Fig. S2.** The coverage of Illumina sequencing reads and contigs in a complete BAC (SHE003C23).


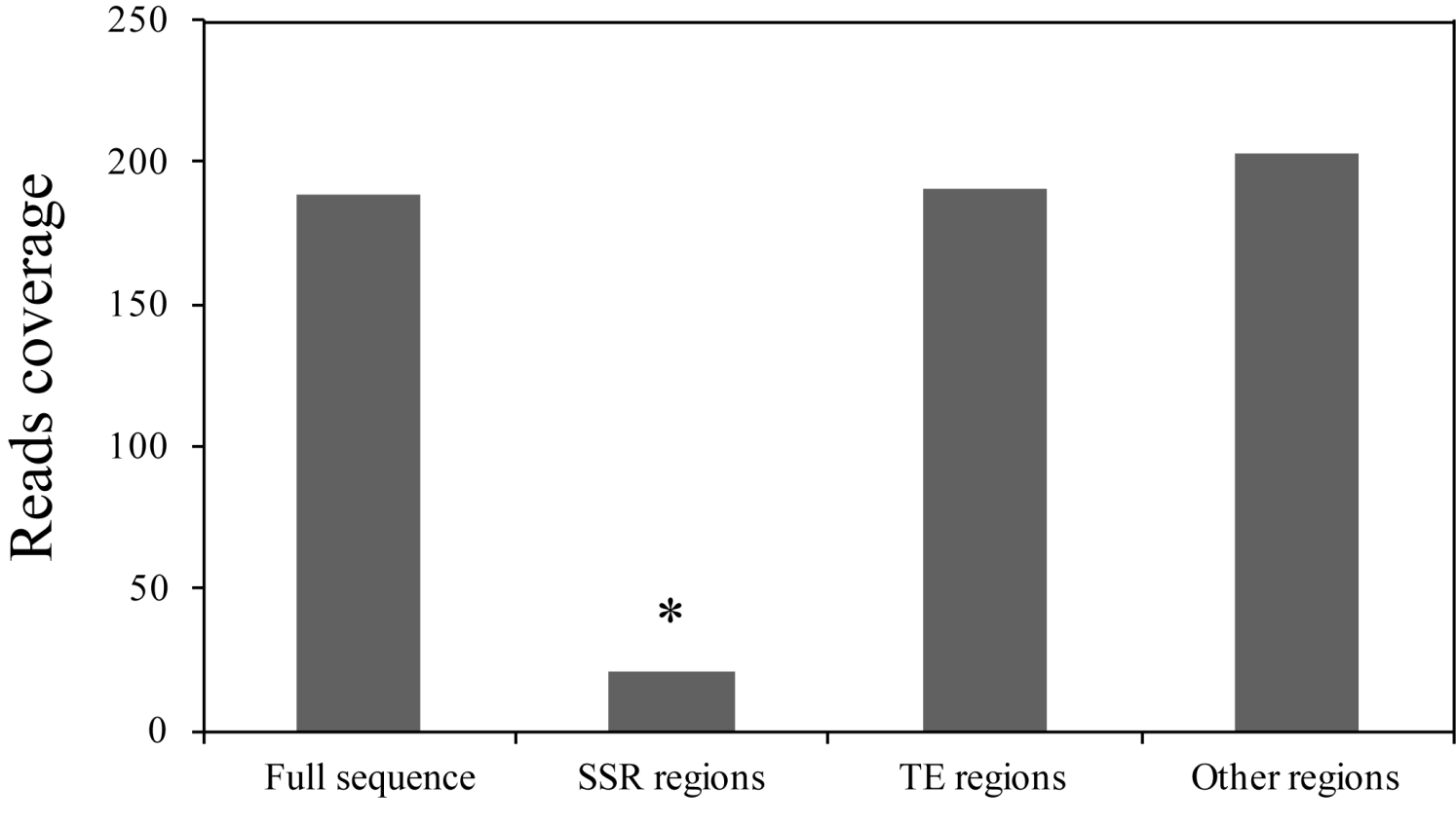


**Fig. S3.** The comparison of reads coverage of various genomic regions.
